# In-depth analysis of T2Bacteria positive results in patients with concurrent negative blood culture: a case series

**DOI:** 10.1186/s12879-020-05049-9

**Published:** 2020-05-07

**Authors:** Markos Kalligeros, Ioannis M. Zacharioudakis, Giannoula S. Tansarli, Katerina Tori, Fadi Shehadeh, Eleftherios Mylonakis

**Affiliations:** 1 Infectious Diseases Division, Warren Alpert Medical School of Brown University, Rhode Island Hospital 593 Eddy Street, POB, 3rd Floor, Suite 328/330, Providence, RI 02903 USA; 2 Division of Infectious Diseases and Immunology, Department of Medicine, NYU School of Medicine, New York, NY USA

**Keywords:** Bacteremia, Bloodstream infection, Sepsis, Septicemia, T2Bacteria assay, T2 magnetic resonance, T2MR, Discordant results

## Abstract

**Background:**

T2Bacteria assay uses T2 magnetic resonance (T2MR) technology for the rapid diagnosis of bacterial bloodstream infections (BSIs). This FDA cleared technology can detect 5 of the most prevalent pathogens causing bacteremia (*Escherichia coli*, *Staphylococcus aureus, Klebsiella pneumoniae*, *Pseudomonas aeruginosa*, and *Enterococcus faecium*). Because the significance of discordant results between the T2Bacteria assay and blood culture (BC) remains a challenge, in this case series we reviewed the medical records of patients who had a positive T2Bacteria test and a concurrent negative BC.

**Methods:**

Among 233 participants, we identified 20 patients with 21 (9%) discordant T2Bacteria-positive/BC-negative (T2+/BC-) results. We classified these results based on clinical cultures and clinical evidence.

**Results:**

When we analyzed these 21 discordant results in-depth, 11 (52.5%) fulfilled criteria for probable BSI, 4 (19%) for possible BSI, and 6 (28.5%) were presumptive false positives. Among the probable/possible BSIs, discordant results were often associated with patients diagnosed with closed space and localized infections [pyelonephritis (*n* = 7), abscess (*n* = 4), pneumonia (*n* = 1), infected hematoma (n = 1), and osteomyelitis (n = 1)]. Also, within the preceding 2 days of the T2+/BC- blood sample, 80% (16/20) of the patients had received at least one dose of an antimicrobial agent which was active against the T2Bacteria-detected pathogen.

**Conclusions:**

In the majority of discrepant results, the T2Bacteria assay detected a plausible pathogen that was supported by clinical and/or microbiologic data. Discrepancies appear to be associated with closed space and localized infections and the recent use of effective antibacterial agents. The clinical significance and potential implications of such discordant results should be further investigated.

## Background

Bloodstream infections (BSIs) are associated with significant morbidity and mortality [1, 2] and timely administration of appropriate antimicrobial therapy is considered critical for improved outcomes [3]. Development of rapid and accurate diagnostic tests, along with their implementation in the everyday clinical practice may significantly decrease the turnaround result time and help with the selection of appropriate antimicrobial therapy [4]. Recently, the US Food and Drug Administration (FDA) cleared the T2Bacteria assay (T2 Biosystems, Lexington, MA). This diagnostic panel is capable of detecting 5 important ESKAPE pathogens (*Escherichia coli*, *Staphylococcus aureus, Klebsiella pneumoniae*, *Pseudomonas aeruginosa*, and *Enterococcus faecium*), by using T2 magnetic resonance (T2MR) directly on whole blood samples. These pathogens represent the majority of healthcare-associated infections and often exhibit multiple drug resistance [5–7].

The diagnostic efficacy of the T2Bacteria assay was determined in a multi-center clinical trial that involved 11 US hospitals and included 1427 patients [8]. The sensitivity and specificity of the T2Bacteria assay were determined based on the results of concurrent blood culture, which is the standard of care for diagnosing BSIs. Per-patient sensitivity and specificity of T2Bacteria for proven BSIs were 90% (95% CI, 76 to 96%) and 90% (CI, 88 to 91%), respectively; while the negative predictive value was 99.7% (1242 of 1246). Importantly, discordant T2Bacteria-positive/blood culture (BC)-negative results (T2+/BC-) represented 10% (146/1427) of all reported tests [8], while the clinical significance of these results is still undetermined.

Since blood culture results may be negative even in cases of severe sepsis [9], and can be affected by factors such as prior antibiotic use [10, 11], our aim was to evaluate the significance of T2+ cases when BC was negative, in an effort to get a better understanding of whether these T2 results were false positives or potentially associated with an infection.

## Methods

### Study design, setting and population of the T2Bacteria clinical tria

This study was a sub-study of a larger, prospective, multi-center clinical trial, which led to the FDA clearance of the T2Bacteria assay. The study population was composed of hospitalized patients (18 years or older), in whom BSI or sepsis was suspected and BC was ordered by the treating physician as per standard of care. After enrollment, aerobic and anaerobic companion BC set (one bottle each) and whole blood samples (for T2Bacteria testing) were collected concurrently and from the same anatomic site. Companion BCs (5–10 ml whole blood per bottle) were performed in accordance with hospital practices and manufacturer’s recommendations. Also, the results of the T2Bacteria panel were not available to the clinicians and did not impact their clinical judgement. Further details about the clinical trial can be found in the original study by Nguyen et al [8].

### Design and data collection of this study

Data of patients who were enrolled in the aforementioned clinical trial, at The Miriam Hospital, Providence, RI, between December 2015 and July 2017, were reviewed. After identifying the patients who had discordant T2+/BC- results, we accessed their medical file and an in-depth analysis of each case was performed. For each patient, we retrospectively reviewed all clinical files and recorded the following: age, gender, pathogen detected by the T2Bacteria assay, positive clinical cultures with the same T2Bacteria-detected pathogen (either from a previous clinical BC or from an extra-blood site, within a time frame of 21 days-as per clinical trial protocol), antibiotics used, history of present illness, radiologic findings, diagnoses and outcomes, including readmission in the following 6 months. In our study site, companion BCs (5 to 10 mL of whole blood per bottle) were performed using the VersaTREK automated detection system (Thermo Fisher Scientific), while bacteria in positive cultures were identified using the VITEK 2 system (BioMérieux). Blood cultures that did not yield an organism were incubated for at least 5 days.

### Definitions

Each discordant T2+/BC- result was classified as probable BSI, possible BSI, or presumptive false positive. More specifically a BC was defined as negative if no bacteria were recovered from a set of BC bottles, while the T2Bacteria result was considered positive if ≥1 of the 5 targeted bacteria were detected. Each medical record was reviewed independently by two investigators (MK, GST).

Discordant T2+/BC- results, were classified as follows:

- *Probable BSI*, if the T2Bacteria-detected microorganism was isolated within 7 days from a clinical BC collected at a different time, or from a clinical culture from an extra-blood site (e.g., abdomen, urine, wound) indicating a plausible cause of infection. In this context, “Day 0” is defined as the day of the T2Bacteria blood sample collection.
- *Possible BSI*, if there was a positive T2Bacteria result in the absence of supporting culture data, provided that the detected bacterium was a plausible cause of the disease (e.g., *E. coli* in a patient with pyelonephritis).
- *Presumptive False Positive,* if none of the above was true.

These definitions were in concordance with those used by Nguyen et al. in their recently published trial [8]. The only difference was the timeframe definition of probable BSI which was stricter in order to avoid representing a new infection. In this regard, we used a 7-day timeframe instead of 21 days.

Patients were considered to be receiving an active antibiotic around the time of testing if they received at least one dose within the 2 days preceding sample collection. For probable BSIs, where at least another culture was positive for the T2Bacteria detected microorganism, culture sensitivities were used to determine if the T2Bacteria-detected microorganism was susceptible to a previously received antibiotic. For possible BSIs and presumptive false positive results, where by definition no isolate was available, the hospital antibiogram was used to determine if the T2Bacteria-detected microorganism was susceptible to the received antibiotic.

Since study participants could have had multiple BCs during their hospital course, and in order to avoid confusion we will refer to the BC collected concurrently with the T2Bacteria blood sample, as companion BC.

## Results

At our study site, 233 patients participated in the original T2Bacteria trial. For the T2 detected microorganisms (*Escherichia coli*, *Staphylococcus aureus, Klebsiella pneumoniae*, *Pseudomonas aeruginosa*, and *Enterococcus faecium*) the results from our site were as follows: 211 patients had a concordant T2−/BC- result, 2 patients had a concordant T2+/BC+ result, 0 patients had a discordant T2−/BC+ result, while 20 out of 233 patients (8.5%) had a discordant T2+/BC- result. For those 20 patients with T2+/BC-, we accessed their medical files and we performed an in-depth analysis. In 1 patient the T2Bacteria assay detected 2 bacteria simultaneously. Therefore, the final analysis describes 21 discordant results. Because of this discrepancy, in the section below we specify if a particular number refers to patients or assay results.

Among patients with a discordant T2+/BC- result, age ranged from 19 to 86 (median age 68 years); 11 were women and 9 were men. In total, 80% (16/20) of the patients had received at least 1 dose of an active antibiotic (based on culture results and/or hospital antibiogram) within the preceding 2 days of the T2+/BC- blood sample. Among 20 patients, 14 were discharged with a diagnosis that included a closed-space or localized infection, such as pyelonephritis (*n* = 7), abscess (*n* = 4), pneumonia (*n* = 1), infected hematoma (*n* = 1), and osteomyelitis (*n* = 1).

For every case, we collected clinical details, including a brief history of presenting illness, clinical culture results, antibiotics used, relevant radiologic findings and discharge diagnoses. As detailed below, among the 21 discordant T2+/BC- results, 11 (52.5%) fulfilled the criteria for a probable BSI, 4 (19%) for a possible BSI and 6 (28.5%) were presumptive false positives.

### Probable BSIs

Probable BSIs comprised more than half of the T2+/BC- discordant results (11 out of 21) (Tables 1 and S1). Although the companion BC turned out negative and led to a discordant T2+/BC- result, the presence of the T2-detected microorganism was supported by at least 1 other clinical culture (median time: − 1 day, range: − 4 to 0 days). These probable BSI cases were due to: *E. coli* (*n* = 6), *S. aureus* (*n* = 3), *K. pneumonia* (*n* = 1), and *P. aeruginosa* (n = 1). A brief clinical vignette summarizing each case can be found on Table 1.

**Table 1:** Clinical and laboratory details of patients with probable BSI

Patient number	Age range, Gender	Brief History of present illness	T2Bacteria result	Other (+) culture with the same T2-detected pathogen within 21 days	Antibiotics used/ Activity against T2 detected bacterium	Radiologic Findings	Discharge Diagnosis
Patient 1	> 65, M	Blood Pressure 80/40 mmHg, heart rate 120/min, fever 38.3 °C. Dysuria and urinary frequency for 5 days PTA	*E. coli*	Blood culture^b^ (−2d) Urine culture (−2d)	CRO, TZP/Yes	CT negative for renal obstruction	ESBL *E coli* bacteremia and pyelonephritis
Patient 2	> 65, F	Fever, chills, nausea, dysuria	*E*. *coli*	Blood culture^b^ (−1d) Urine culture (−1d)	CRO /Yes	–	Pyelonephritis with *E coli* bacteremia
Patient 3	50–64, F	Nausea, vomiting, burning with urination, abdominal and flank pain, fever	*E*. *coli*	Urine culture (0d)	CRO, TZP/Yes	–	Pyelonephritis due to *E. coli*
Patient 4	> 65, F	Chills, rigors, dyspnea, dysuria	*E*. *coli*	Urine culture (0d)	CRO /Yes	CT: bilateral pyelonephritis	Bilateral *E coli* pyelonephritis
Patient 5	> 65, F	Fever, nausea, vomiting, abdominal pain and dark foul-smelling urine for 1 week.	*E*. *coli*	Urine culture (−1d)	CRO, TZP /Yes	CT: 4 mm obstructing calculus, mild hydroureteronephrosis	Pyelonephritis due to *E coli*
Patient 6	> 65, M	Fever, dysuria and sepsis.	*E*. *coli*^a^	Urine culture (−1d)	CRO /Yes	CT: Right sided Pyelonephritis	Pyelonephritis due to *E coli*
Patient 7	> 65, F	Weakness, productive cough, fever, nausea and vomiting. Symptoms started 5 days PTA.	*S. aureus*	Blood culture^b^ (−4d)	VAN, CRO, AZM/ Yes	CXR with right lower lobe pneumonia	Influenza B, superimposed *S. aureus* pneumonia
Patient 8	50–64, M	Left thigh abscess and sepsis. Had a previous visit for left thigh abscess 1 month ago, which was incised.	*S*. *aureus*	Wound incision and drainage culture (−2d) with MRSA	VAN, SAM/Yes	U/S: Subcutaneous edema. No drainable abscess	Left thigh abscess/ MRSA wound infection
Patient 9	> 65, M	Left third finger abscess and fever for 8 days. Cellulitis/abscess in left third finger.	*S*. *aureus*	Blood culture^b^ (−11d) Finger abscess (−2d)	VAN, TZP/Yes	*–*	Finger osteomyelitis, Bacteremia due to *S*. *aureus*
Patient 10	18–49, M	Flank pain, chills, dysuria, hematuria. Initially admitted with obstructing mid ureteral calculus and UTI. Underwent urgent right ureteral stent placement.	*K. pneumonia*	Urine culture (−3d)	AMP, CRO /Yes	CT: Multifocal abscess formation in the right kidney	Obstructing mid-ureteral calculus with UTI and multifocal kidney abscesses.
Patient 11	> 65, M	Fever, shaking chills and nausea. History of recurrent UTIs and benign prostatic hyperplasia requiring self-catheterization with Foley catheter.	*P. aeruginosa*	Urine culture (−1d)	VAN, TZP/Yes	–	Complicated UTI due to *P. aeruginosa*

^a^T2Bacteria detected 2 targeted organisms in the sample of this patient. Please also see Table 3. ^b^Refers to a previous blood culture, not the blood culture that was taken at the same time with the T2 blood sample

*AMC* Amoxicillin-clavulanic, *AZM* Azithromycin, *CFZ* Cefazolin, *CIP* Ciprofloxacin, *CRO* Ceftriaxone, *CT* Computed Tomography, *F* Female, *M* Male, *MEM* Meropenem, *MRSA* Methicillin Resistant *S. aureus* SAM Ampicillin-Sulbactam, *PTA* prior to admission, *TZM* Piperacillin-tazobactam, *U/S* Ultrasound, *UTI* Urinary Tract Infection, *VAN* Vancomycin

In all 11 T2+/BC- results classified as probable BSIs, the patient had already received antibiotics for a mean of 2.5 days prior to the T2 blood draw (Table 1). The susceptibility results from the supporting clinical cultures showed that in all of these cases the antibiotics were active against the pathogen detected by the T2Bacteria assay. Also, antecedent antibiotic use is partly explained by the fact that in 8 of these 11 patients (73%), the companion T2 BC was chronologically the second set of BC to be drawn during the same hospitalization course, therefore the patient was already receiving antibiotics (Table S1).

All patients were discharged without subsequent readmission, except for 1 case (patient n.7) who was later readmitted due to bacteremia with the previously detected T2Bacteria microorganism. In this case a BC set was received on hospital day 1 and the patient received therapy for suspected pneumonia. On day 4, the first set of BC was still negative and the T2Bacteria sample and companion BC were obtained. On day 5, 1 bottle from day 1 BC yielded *S. aureus* growth but the patient was discharged on oral ampicillin/clavulanate. She was readmitted 2 days later when the second bottle of the same set of day 1 BC turned out positive as well. T2Bacteria was positive for *S. aureus* since Day 4 (see also Tables 1 and S1).

Regarding the site of infection, 7 patients were discharged with the diagnosis of pyelonephritis, and 1 each with pneumonia, thigh abscess, osteomyelitis, and kidney abscess. In all cases the causative microorganism was eventually isolated and it was the same that the T2Bacteria had detected. No other plausible microorganisms were detected from clinical cultures. As shown in Table S1, the antibiotic choice of these patients was made based on the clinical picture on admission and it was later tailored based on the sensitivity results of the positive clinical culture.

### Possible BSIs

We classified 4 results as possible BSI and each one of them represents a different patient (Tables 2 and S2). In these cases, there was no supporting clinical culture evidence of the T2Bacteria assay result. However, the T2Bacteria-detected pathogen seemed as a plausible cause of the discharge diagnosis. More specifically, T2Bacteria detected *S. aureus* in the bloodstream of a patient who was an active injection drug user. The patient presented with cough and fever, and was later discharged with the diagnosis of pneumonia. T2Bacteria also detected 2 cases of *P. aeruginosa.* The first was detected in a patient who had diverticulitis with micro-perforations on imaging. The second was detected in a patient with infected hematoma 4 days after appendectomy, in whom drainage of his hematoma yielded a polymicrobial infection. Finally, T2Bacteria detected *E. coli* in a patient with fever, nausea, vomiting and severe sepsis, who was on treatment with trastuzumab (Herceptin®) and discharged without a relevant diagnosis.

**Table 2:** Clinical and laboratory details of patients with possible BSI

Demographics	Age range, Gender	Brief History of present illness	T2Bacteria result	Other (+) culture with pathogen different than T2-detected within 21 days	Antibiotics used/Activity against T2 detected bacterium (% susceptibility)	Radiologic Findings	Discharge Diagnosis
Patient 12	18–49, F	Chest pain, fever, hypoxemia, and tachycardia. Active Cocaine/Heroin IV user, HCV, recent dental surgery.	*S*. *aureus*	No	VAN, AZM, TZP/ (VAN-100%)	CT: c/w pneumonia. TTE was negative	Pneumonia
Patient 13	> 65, F	Sudden onset left sided abdominal pain, nausea, vomiting.	*P. aeruginosa*	No	CIP, MTZ / (CIP-85%)	i) CT (day 1): Acute uncomplicated diverticulitis ii) CT (day 3): Minimally complicated diverticulitis with micro-perforation, as well as new secondary enteritis and small bowel obstruction	Diverticulitis
Patient 14	18–49, F	Fever, tachycardia, nausea, vomiting. Diagnosed with breast cancer, on Trastuzumab (had chest port site).	*E*. *coli*	BC from port site yielded CoNS (−1d) (considered contaminant)	VAN, FEP / (FEP-98%)	–	Sepsis without clear source identified
Patient 15	18–49, M	Abdominal pain, fever, nausea after appendectomy (Postoperative day 4). Reported a sharp RLQ pain and ecchymosis surrounding his incisions.	*P. aeruginosa*	Culture of drainage from right lower quadrant collection yielded *E. coli* and *Clostridium* species	TZP / (TZP-91%)	CT: Right lower quadrant collection compatible with a postoperative hematoma with possible superinfection	Infected postoperative hematoma

*AZM* Azithromycin, *CIP* Ciprofloxacin, *CFZ* Cefazolin, *CoNS* Coagulase-negative Staphylococcus, *CT* Computed Tomography, *c/w* compatible with, *F* Female, *FEP* Cefepime, *M* Male, *MEM* Meropenem, *MTZ* Metronidazole, *TTE* Transthoracic Echocardiogram, *TZP* Piperacillin-Tazobactam, *UTI* Urinary tract infection, *VAN* Vancomycin

Prior to the blood draw, all patients had received an antibiotic for which the T2Bacteria detected microorganism was at least 85% susceptible based on hospital antibiogram (susceptibility available on Table 2). In all patients the outcome was discharge without subsequent readmission, except for patient n.13 who was readmitted with diverticulitis after 15 days. At that time, computed tomography (CT) showed sigmoid diverticulitis with small abscess formation which was not amenable to percutaneous drainage and BC was again negative.

### Presumptive false-positive results

In total 6 T2+/BC- results were defined as presumptive false-positives (Tables 3 and S3). For patient n.6, the T2Bacteria was positive for both *E. coli* and *P. aeruginosa* (discussed below). After reviewing the electronic medical records of these patients, the T2Bacteria-detected pathogens did not seem to correlate with either the discharge diagnosis or any laboratory culture. Interestingly, 2/6 (33%) of these patients had received an antibiotic for which the T2Bacteria-detected pathogen was susceptible based on hospital antibiogram. Moreover, patient n.18, in whom T2Bacteria detected *E. coli* in his blood, had a recent history of multiple urinary tract infections (UTIs). However, due to lack of urinary symptoms and his unrelated presenting symptom, no urine culture was performed. Finally, as noted above, in patient n.6, T2Bacteria was positive for both *E. coli* and *P. aeruginosa*, while the discharge diagnosis was pyelonephritis with positive urine culture for *E. coli*. Thus, the *E. coli* result was classified as probable BSI (Table 1), while *P. aeruginosa* as false positive because the chance of concurrent infection was considered low. The outcome in all of these cases was discharge without readmission.

**Table 3:** Clinical and laboratory details of patients with presumptive false positive results

Demographics	Age range, Gender	Brief History of present illness	T2Bacteria result	Other (+) culture with other than T2-detected pathogen within 21 days	Antibiotics used/Activity against T2 detected bacterium	Relevant Radiologic Findings	Discharge Diagnosis
Patient 16	> 65, F	Nausea, vomiting, imbalance and blurry vision. No signs of infection. A TTE performed on day 4 showed a mobile echo-density on the aortic valve which was consistent with Lambl’s excrescence or vegetation and prompted BCs.	*E*. *coli*	No	None	MRI brain: Large acute infarct in the right cerebral hemisphere.	Stroke
Patient 17	50–64, F	Patient with abdominal pain and melena	*E*. *coli*	*Cutibacterium acnes*	None	Abdomen CT: pancreatitis with multiple pseudocysts	Alcohol-induced acute pancreatitis with pancreatic pseudocyst.
Patient 18	50–64, M	﻿2 days PTA patient visited the ED for traumatic shoulder injury and BC was received. Admitted because BC yielded CoNS.	*E*. *coli*	No	None	–	Musculoskeletal shoulder injury
Patient 19	18–49, M	HIV positive on HAART treatment. (last CD4 was 450 cells/mm^3^) presents febrile (up to 38.8 °C) after status epilepticus.	*P. aeruginosa*	No	CRO, VAN/ No	Normal CT and MRI of the brain	Seizure Disorder
Patient 20	18–49, F	3 days of diffuse body rash, fever (40.4 °C) and headache.	*P. aeruginosa*	No	FEP, VAN, DOX/ Yes (FEP-96%)	–	Undiagnosed/ Possible Q Fever, Parvovirus B19, Toxoplasmosis
Patient 6	> 65, M	Fever, dysuria, sepsis	*P. aeruginosa*^a^	No	CIP/ Yes (CIP-85%)	–	Pyelonephritis

^*a*^*T2Bacteria detected 2 targeted organisms in the sample of this patient. Please also see* Table 1

*BC* Blood Culture, *CIP* Ciprofloxacin, *CRO* Ceftriaxone, *CoNS* Coagulase-negative Staphylococcus, *CX* culture, *DOX* Doxycycline, *F* Female, *FEP* Cefepime, *HAART* Highly active antiretroviral therapy, *M* Male, *MSSA* Methicillin susceptible *S. aureus*, *PTA* prior to admission, *T2/BC* T2 sample and the “companion” blood culture, *TTE* Transthoracic echocardiogram, *UCX* urine culture, *VAN* Vancomycin

## Discussion

The T2Bacteria panel is a new test for the rapid diagnosis of BSI caused by 5 of the most prevalent bacterial pathogens. In this study we reviewed in detail 20 patients with 21 discordant T2+/BC- results, in an effort to evaluate the robustness of T2Bacteria positive results, in the context of a concurrent negative BC. We found that most of the discordant results (71%) were due to probable or possible BSIs and in all of these cases the patients had received an active antibiotic against the T2Bacteria-detected microorganism. Localized infections, as well as antibiotic use before the T2Bacteria sample, might be associated with discordant results in patients with probable or possible BSIs. Importantly, even though in most cases the positive T2Bacteria result was supported by clinical information, the clinical significance of these results and the need to treat solely based on a T2Bacteria positive result needs to be studied.

The interpretation of a new assay poses a major challenge in diagnostic accuracy studies [12]. In the T2Bacteria trial, in order to estimate accuracy, the authors assumed that the reference standard, i.e. the BC, is 100% sensitive and specific for bacteremia diagnosis [8]. However, the sensitivity of a BC is suboptimal and significantly hampered in certain conditions, such as the antecedent antibiotic use [13]. In this context, the detailed review of clinical circumstances is needed in order to appreciate the clinical significance of a positive result. In our review, the finding that the majority of discordant results were either probable or possible BSIs underlines that a positive T2Bacteria assay result might have had an even closer association with the pathogen involved.

Besides investigating the validity of T2+ results in the context of a negative BC, our analysis provides a better understanding of the different factors which likely affected these results. Interestingly, 14 out of 20 patients with discordant results were later discharged with a diagnosis of closed-space or localized infection. Such infections can cause intermittent bacteremia, thus constituting detection by a BC challenging [14, 15]. However, T2Bacteria may have an increased capability of pathogen detection in this context, due to its ability to identify bacterial DNA, which might have persisted in patient’s bloodstream after a period of intermittent bacteremia [8]. From a clinical perspective, recognizing the pathogen associated with closed space and localized infections early in the course of hospitalization and without the need of a surgical procedure, may be proven significant, since early and effective source control of infection can play a pivotal role in those patients' outcome [16].

Concurrent antibiotic use also played a significant role in the majority of T2+/BC- results. In the study by Nguyen et al. [8], apart from collecting the T2Bacteria sample concurrently with the companion BC, no other strict timing regulation was imposed. Obtaining a BC during antibiotic therapy is associated with a significant decrease of pathogen detection [17, 18]. A recent study by Scheer et al. reported a loss of 23% in BC positivity of patients with sepsis who had already received antibiotics [13]. T2Bacteria assay allows the detection of bacterial cell-associated DNA even in the presence of substances that inhibit cell growth [19] and is less likely to be impacted by antecedent antibiotic use [8, 20].

In addition to the clinical significance of discordant results, careful selection of the optimal time to perform the T2Bacteria test has yet to be defined. Performing T2Bacteria as a part of the initial diagnostic work up along with the BC, could provide the clinician with a higher cumulative sensitivity for bacteremia detection. Also, identification of the causative organism by the T2Bacteria assay, even without antibiotic susceptibilities, could help clinicians streamline and adjust the empiric therapy, based on detection of organisms with unique susceptibility profiles or high local resistance rates [21].

The present study has some notable limitations. First, we investigated in depth only the discordant results from our study site. Second, since the clinicians were not informed of the T2Bacteria results during the conduction of the trial, our study cannot provide information on the clinical significance of a positive T2Bacteria result. Finally, the assay is capable of diagnosing only 5 pathogens and cannot replace the BCs, which will be required for the detection of other pathogens as well as for susceptibility testing. Consequently, this is posing a question regarding the excess costs and utilization of healthcare resources [22].

## Conclusions

In conclusion, based on a single-center experience, in the majority of discrepant cases a positive T2Bacteria assay was associated with a plausible pathogen that was supported by clinical and/or laboratory data. On the other hand, almost one third of the cases yielded false positive results highlighting the need for molecular testing stewardship and careful clinical interpretation of results. The clinical significance, cost-effectiveness, and optimal timing of the assay should be studied further, and, in these studies, the evaluation of discordant results should focus on patients already receiving antibiotics and those with a potential closed-space or localized infection.

## Supplementary information

**Additional file 1 Table S1:** Sequence of clinical cultures and antibiotics used during the hospitalization course of patients with probable BSI. **Table S2:** Sequence of clinical cultures and antibiotics used during the hospitalization course of patients with possible BSI. **Table S3:** Sequence of clinical cultures and antibiotics used during the hospitalization course of patients with presumptive false positive results

## Data Availability

The datasets generated and/or analysed during the current study are not publicly available due to HIPAA restrictions but are available from the corresponding author on reasonable request.

## Abbreviations

T2MR: T2 magnetic resonance
BSI: Bloodstream infection
BC: Blood culture
T2+/BC-: T2Bacteria-positive / blood culture negative
FDA: US Food and Drug Administration
