# Supplementary material for: In-depth analysis of T2Bacteria positive results in patients with concurrent negative blood culture: a case series
Source: BMC Infect Dis. 2020 May 7;20:326. doi: 10.1186/s12879-020-05049-9 (PMC7206677; doi:10.1186/s12879-020-05049-9)
Supplement: Supplementary file 1 — Additional file 1 Table S1: Sequence of clinical cultures and antibiotics used during the hospitalization course of patients with probable BSI. Table S2: Sequence of clinical cultures and antibiotics used during the hospitalization course of patients with possible BSI. Table S3: Sequence of clinical cultures and antibiotics used during the hospitalization course of patients with presumptive false positive results [file 12879_2020_5049_MOESM1_ESM.docx]

**Supplementary**

**Table S1:** Sequence of clinical cultures and antibiotics used during the hospitalization course of patients with probable BSI

|  | Clinical culture performed OR clinical culture result on that day / (Antibiotics used on that day) | | | | | | |
| --- | --- | --- | --- | --- | --- | --- | --- |
|  | **Day 1** | **Day 2** | **Day 3** | **Day 4** | **Day 5** | **Day 6** | **Day 7** |
| Patient 1 | BC**^1^**, UCX  (TZP, VAN) | Both BC**^1^** and UCX yielded *E. coli*  (TZP, VAN) | **T2/BC^2^**,  (CRO) | UCX antibiogram with ESBL *E. coli*  (MEM) | (MEM) | (MEM) | (MEM)  Discharged without antibiotics |
| Patient 2 | BC^1^, UCX  (CRO) | BC**^1^** yields GN rods,  UCX yields *E. coli*,  **T2/BC^2^**  (CRO) | UCX antibiogram available  (CRO) | (CRO, CIP) | Discharged on CIP P.O. for 7 days | - | - |
| Patient 3 | UCX, **T2/BC^1^**  (TZP) | (TZP) | (TZP)  Discharged on CIP for 7 days | - | - | - | - |
| Patient 4 | BC**^1^**, UCX  (MEM, VAN, TZP, CRO) | UCX yields *E. coli*,  **T2/BC^2^,**  (MEM) | UCX antibiogram available  (MEM, AMC) | Discharged on AMC for 7 days | - | - | - |
| Patient 5 | UCX  (CRO) | UCX yields *E. coli*,  **T2/BC^1^**  (TZP) | (TZP) | UCX antibiogram available  (TZP) | (CIP) | Discharged on CIP P.O. for 8 days | - |
| Patient 6 | BC**^1^**, UCX  (CRO, AZM, CIP) | UCX yields *E. coli*,  **T2/BC^2^,**  (CRO, AZM) | UCX antibiogram available  (CRO) | (CRO) | Discharged on LVX for 14 days | - | - |
| Patient 7 | BC**^1^**  (CRO, AZM, VAN) | (CRO, AZM) | **T2/BC^2^**  (CRO, AZM) | BC**^1^** yields GP cocci,  Discharged on AMC for 2 days | - | - | - |
| Patient 8 | BC**^1^,** Wound drainage culture  (VAN, SAM) | BC yields CoNS  (VAN, SAM) | Wound drainage yields MRSA,  **T2/BC^2^**  (VAN, SAM) | (VAN) | (VAN) | Discharged on CLI P.O. for 7days | - |
| Patient 9 | BC**^1^,** Finger Abscess CX  (TZP, VAN) | Tendon CX  (TZP, VAN) | Abscess CX yields MSSA**, T2/BC^2^**  (TZP, VAN) | Tendon CX yields MSSA, Antibiogram Available  (CFZ) | (CFZ) | Finger amputation. Bone CX  (CFZ) | Discharged on CFZ with PICC line. |
| Patient 10 | UCX  (CRO, AMP) | (CRO, AMP) | (CRO, AMP) | **T2/BC^1^,**  (CRO, AMP) | Nephrectomy  (CRO, AMP, CFZ) | (CRO, AMP) | Day 7- Day 13  (CRO)  Discharged on Day 13 on no antibiotics |
| Patient 11 | BC**^1^**, UCX  (TZP, VAN) | UCX yields *P. aeruginosa*,  **T2/BC^2^**  (TZP) | UCX antibiogram  (TZP) | (TZP) | (TZP) | Discharged on IV FEP for 8 days. | - |
| The number next to the BC refers to the order. E.g. BC^1^ indicates BC was the first BC during that hospitalization  AMC: Amoxicillin-Clavulanic, AZM: Azithromycin, BC: Blood Culture, CFZ: Cefazolin, CIP: Ciprofloxacin, CLI: Clindamycin, CoNS: Coagulase-negative Staphylococcus, CRO: Ceftriaxone, CX: culture, FEP: Cefepime, GN: gram negative, GP: gram positive, LVX: Levofloxacin MEM: Meropenem, MSSA: Methicillin susceptible *S. aureus,* PICC: Peripherally inserted central catheter, P.O: Per os, SAM: Ampicillin-Sulbactam, TZM: Piperacillin-Tazobactam, T2/BC: T2 sample and “companion” blood culture, UCX: urine culture, VAN: Vancomycin | | | | | | | |

**Table S2:** Sequence of clinical cultures and antibiotics used during the hospitalization course of patients with possible BSI

|  | Clinical Culture Performed OR clinical culture result on that day / (Antibiotics used on that day) | | | | | | |
| --- | --- | --- | --- | --- | --- | --- | --- |
|  | **Day 1** | **Day 2** | **Day 3** | **Day 4** | **Day 5** | **Day 6** | **Day 7** |
| Patient 12 | **T2/BC^1^**  (TZP, VAN, AZM) | (TZP, VAN) | (TZP, VAN) | (TZP, VAN) | (TZP, VAN) | (TZP, VAN) | (TZP, VAN)  Discharged on CIP for 2 days |
| Patient 13 | UCX  (CIP, MTZ) | (CIP, MTZ) | **T2/BC^1^**  (CIP, MTZ) | (CIP, MTZ) | (CIP, MTZ) | (CIP, MTZ) | Discharged on CIP MTZ for 7 days |
| Patient 14 | BC**^1^**, UCX  (FEP) | **T2/BC^2^**  (FEP, VAN) | (FEP, VAN) | Discharged without antibiotics | - | - | - |
| Patient 15 | drainage CX  (TZP) | CX yielded *E. coli*, **T2/BC^1^**  (TZP) | (TZP) | Discharged on AMC for 10 days | - | - | - |
| The number next to the BC refers to the sequence. For example, BC^1^ indicates BC was the first BC during that hospitalization.  BC: Blood Culture, CX: culture, MSSA: Methicillin susceptible *S. aureus*, T2/BC: T2 sample and the “companion” blood culture, UCX: urine culture  AMC: Amoxicillin-Clavulanic AZM: Azithromycin, CRO: Ceftriaxone, CIP: Ciprofloxacin, CFZ: Cefazolin, FEP: Cefepime, MEM: Meropenem, VAN: Vancomycin, TZP: Piperacillin-Tazobactam | | | | | | | |

**Table S3:** Sequence of clinical cultures and antibiotics used during the hospitalization course of patients with presumptive false positive results

|  | Clinical Culture Performed OR clinical culture result on that day / (Antibiotics used on that day) | | | | | | |
| --- | --- | --- | --- | --- | --- | --- | --- |
|  | **Day 1** | **Day 2** | **Day 3** | **Day 4** | **Day 5** | **Day 6** | **Day 7** |
| Patient 16 | - | - | - | **T2/BC^1^** | Discharge | - | - |
| Patient 17 | BC^1^ | - | **^-^** | - | BC^1^ yielded *P. acnes*,  **T2/BC^2^**  (TZP, VAN) | (TZP, VAN) | Day 13-Discharged without antibiotics |
| Patient 18 | **T2/BC^1^** | Discharge | - | - | - | - | - |
| Patient 19 | BC^1^, LP  (CRO, VAN) | (CRO, VAN) | BC^2^  (CRO, VAN) | (CRO, VAN) | **T2/BC^3^**  Discharged without antibiotics | - | - |
| Patient 20 | BC^1^, UCX, LP  (FEP, VAN, DOX) | **T2/BC^2^**  (FEP, VAN, DOX) | (FEP, VAN, DOX) | (DOX) | (DOX) | Discharged on DOX for 15 days | - |
| The number next to the BC refers to the sequence. For example, BC^1^ indicates BC was the first BC during that hospitalization.  BC: Blood Culture, CIP: Ciprofloxacin, CRO: Ceftriaxone, CX: culture, DOX: Doxycycline, FEP: Cefepime, LP: Lumbar Puncture, T2/BC: T2 sample and the “companion” blood culture, TZP: Piperacillin-Tazobactam, UCX: urine culture, VAN: Vancomycin | | | | | | | |
